# The role of AFB microscopy training in improving the performance of laboratory professionals: analysis of pre and post training evaluation scores

**DOI:** 10.1186/1472-6963-13-392

**Published:** 2013-10-07

**Authors:** Pawlos Reji, Getachew Aga, Gemeda Abebe

**Affiliations:** 1 TB CARE I/ Management Sciences for Health, Addis Ababa, Ethiopia; 2 Oromia Health Bureau, Addis Ababa, Ethiopia; 3 Department of Medical Laboratory Sciences and Pathology, Jimma University, Jimma, Ethiopia

**Keywords:** Tuberculosis, AFB, Refresher training, Scores

## Abstract

**Background:**

Tuberculosis (TB) remains major cause of morbidity and mortality due to any one of infectious agent worldwide. In low income countries, Ziehl-Neelsen sputum smear microscopy is the only cost-effective tool for diagnosis and monitoring of patients on treatment. In order to have efficient AFB microscopy centers, it is imperative to have continuous refresher training for laboratory professionals and strong External Quality Assessment (EQA) system). However, very little data exists as to the effect of in-service training on performance of laboratory personnel in Ethiopia.

The objective of this study was to investigate the role of AFB microscopy refresher training on the performance of laboratory professionals.

**Methods:**

A cross-sectional retrospective study was conducted to appraise theoretical and practical performance of laboratory professionals before and after AFB microscopy training. Theoretical assessment was based on standard questions while practical assessment was based on smear reading of 10 standard slides. Data on eight rounds of a five days training at Adama regional laboratory on AFB microscopy in 2009 was obtained and analyzed using SPSS 16.0 statistical software.

**Result:**

The pre-training mean score of the theoretical knowledge and practical skills were 61.8% and 75.7%, respectively. The post training mean scores were 84.2% and 89.2% for theoretical knowledge and practical skills, respectively. The increase in mean score of both theoretical and practical assessment was statistically significant (p < 0.0001). Post training mean score of theoretical knowledge was higher among diploma holders trainees than the BSc degree counter parts (p = 0.001). The mean scores on practice before and after training was dependent on participation in previous AFB microscopy trainings (p < 0.0001). Proportions of trainees with both major and minor errors were found to decrease after they were trained. Trainees who have had previous training were found to commit less errors than those who were not participated in previous training (p < 0.0001).

**Conclusion:**

Training has improved theoretical and practical performance of laboratory professionals. Pre-placement and continuous training irrespective of lab professionals qualification and service year and sustainable EQA are highly recommended to ensure quality of AFB microscopy service.

## Background

Tuberculosis remains major cause of morbidity and mortality due to any one of infectious agent worldwide. It is estimated that one third of the worlds population is infected with *M. tuberculosis* and this result in an estimated eight million new cases annually [1]. Among these new cases 2 to 3 million deaths occur annually [2]. Most of the tuberculosis cases and deaths are reported in developing countries [1]. As per the World Health Organization (WHO) report of 2011, Ethiopia ranks seventh among the 22 tuberculosis high burden countries. According to the report, the estimated prevalence and incidence of all forms TB in Ethiopia was 394 and 261/100,000 population, respectively [3].

Case detection through quality assured bacteriology is an essential element of the WHO STOP TB Strategy [4]. In low income countries, Ziehl-Neelsen sputum smear microscopy is the only cost-effective tool for diagnosing patients with infectious tuberculosis and to monitor their progress in treatment [5]. It yields timely results but the sensitivity is low as compared fluorescent microscopy and culture [6,7].

In order to have an efficient TB microscopy centers, it is imperative to have strong External Quality Assessment (EQA) in which laboratory results are checked by an external agency. Moreover, refresher training for laboratory professionals involved in Acid Fast Bacilli (AFB) microscopy at peripheral health facilities is important [8-10]. EQA which consists of blind rechecking, panel/proficiency testing and onsite supervision is important to identify errors so that corrective actions can be taken to improve the overall performances of microscopy centers [11]. Training of professionals along with sustainable EQA is important to improve the technical competency of laboratory professionals in every aspects of AFB microscopy [12,13].

Studies in Africa and other different parts of the world have shown that effective and sustainable EQA and training programs are significant in improving the performance of AFB microscopy centers [10,14,15]. In Ethiopia multiple trainings have been conducted but most of the training data were not analyzed to see the overall effect of training on performance of the professionals that can be measured in terms of scores on theoretical and practical assessments. Therefore, the aim of present study was to appraise the performance of laboratory professionals before and after they were trained in AFB microscopy in Oromia region.

### Significance of the study

Despite many AFB microscopy trainings, investigation on training data is not commonly practiced in Ethiopia and other countries. Investigation of training data is important as it can provide information on performance of trainees so that appropriate interventions can be planed. Therefore the finding of this study will alert trainers to plan for enhanced quality of AFB microscopy trainings and policy makers in tuberculosis control program to give attention to continuous on job AFB microscopy training towards enhanced case detection and better control of tuberculosis.

### Ethical considerations

Before the commencement of the investigation, official approval was obtained from the ethical clearance committee of Oromia region health bureau (IRB number BEFO/BTFH/1-8/2066). Adama regional laboratory was also officially communicated and permission was obtained to get trainees’ information from the training data base.

## Methods

Retrospective investigation was conducted in December 2011 to assess effect of AFB microscopy training on performance of laboratory professionals. Data on eight rounds of a five days training for 316 trainees on AFB microscopy in 2009 was obtained and analyzed. All these trainees were enrolled at different times in the eight rounds of training and their evaluation was based on their score on pre and post training assessment on theory and practice. Both theoretical and practical assessments were corrected by the trainers who were senior medical laboratory professionals with at least Bachelors degree in medical laboratory technology and minimum of 5 years of service. All of them were certified with training of trainers (TOT) in AFB microscopy.

Theoretical evaluation was based on standard multiple choice questions corrected out of hundred. Practical assessment was based on smear reading on a set of 10 stained panel slides that were prepared and graded as per the standard procedure [11]. Each trainee was provided with 10 stained slides and the reading was corrected out of 100 in which 10 point was given for correct reading, 5 point for Quantification Error (QE) and 0 point for any type of false reading (Low False Positive, High False Positive, Low False Negative or High False Negative) as per the scoring system of proficiency in reading [16]. The overall evaluation system was based on the following table (Table 1).

**Table 1:** Evaluation system for AFB microscopy errors

**Result of trainees**	**True results of standard slides**				
	**Negative**	**1-9 AFB/100 field**	**1+**	**2+**	**3+**
Negative	Correct	LFN	HFN	HFN	HFN
1–9 AFB/100 field	LFP	Correct	Correct	QE	QE
1+	HFP	Correct	Correct	Correct	QE
2+	HFP	QE	Correct	Correct	Correct
3+	HFP	QE	QE	Correct	Correct

LFP- low false positive, HFP- high false positive, LFN- low false negative, HFN- high false negative, QE- quantification error.

Using data collection format, trainees’ score on pre and post training theoretical and practical assessments as well as information on their characteristics including sex, qualification, service year, participation in previous training and their facility type was collected from the training data base of Adama regional laboratory. Data was entered and analyzed using SPSS statistical soft ware (version 16) at a statistical significance of p < 0.05. The mean theoretical and practical scores as well as error types with their rates on smear reading before and after training were determined. Mean score before and after training was compared using paired T test. The effect of trainees’ characteristics on their theoretical and practical scores as well as error rates before and after training was statistically tested using logistic regression analysis.

## Results

### Characteristics of trainees

**Figure 1:**
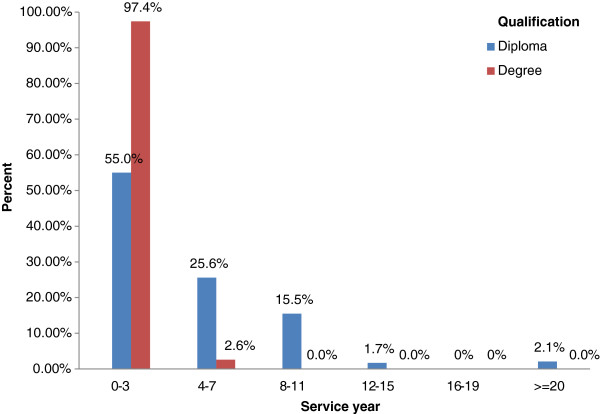
Distribution of trainees by service year and qualification, Adama regional laboratory, 2009.

Out of 316 trainees, 259 (82%) were males, 238 (75.3%) with qualification of diploma, 270 (65.5%) with service year ranging from 0 to 3 years, 169 (53.5%) did not participate in similar previous trainings and 236 (74.3%) were from government health institutions. No trainee with BSc degree had service year of eight or more. Analysis of training data has shown that more than half of the trainees enrolled in 2009 have not been participated in previous trainings (Table 2). Out of total trainees with qualification of BSc degree, 97.4% and 2.6% had service year of 0–3 and 4–7, respectively (Figure 1).

**Table 2:** Frequency distribution of characteristics of trainees, Adama regional laboratory, 2009

**Characteristics**		**Frequency n (%)**
**Sex**	Male	259(82.0)
	Female	57(18.0)
**Qualification**	Diploma	238(75.3)
	Degree	78(24.7)
**Service year**	0–3	207(65.5)
	4–7	63(19.9)
	8–11	37(11.7)
	12–15	4(1.3)
	> = 20	5(1.6)
**Previous training**	Yes	147(46.5)
	No	169(53.5)
**Health institution**	Government	236(74.3)
	Private	80(25.7)

### Theoretical and practical scores of trainees

Data on pre training evaluation have shown that the mean score of the trainees in the theoretical assessment was 61.8% with minimum score of 20%, maximum score of 100%. Pre training practical assessment has shown that the mean, minimum and maximum scores of trainees were 75.7%, 20%, 100%, respectively. Analysis of post training evaluation has shown that the mean, minimum and maximum scores of trainees in theory were 84.2%, 40%, and 100%, respectively. Data on post training evaluation revealed that the mean, minimum and the maximum, scores were 89.3%, 50% and 100%, respectively in practical performance. Post training mean score of trainees in theory and practice was significantly increased (P < 0.0001).

### Theoretical and practical scores by characteristics of trainees

Trainees’ characteristics were investigated to identify their effect on mean score of theoretical knowledge before and after training. The pre training theoretical mean score was not affected by any of the investigated trainees’ characteristics but post training assessment has shown that the mean score of theory was higher among diploma holder trainees than the degree counter parts (p = 0.001) (Table 3). Pre and post training mean score in practice was not dependent on trainee’s sex, qualification and service year (P > 0.05). But it was observed that both pre and post training mean scores in practice was higher among trainees who have had previous training than those who have had no such training (P < 0.0001). It was also found that the mean score of practice after training was higher among participants from private health institutions than the government counterparts (P < 0.011) (Table 4).

**Table 3:** Pre and post training theoretical mean score on smear reading by different characteristics of trainees, Adama regional laboratory, 2009

**Trainees’ characteristics**		**Pre training theoretical mean score in %**	**SD**	**P-value**	**Post training theoretical mean score in %**	**SD**	**P-value**
**Sex**	Male	61.8	15.3	0.857	83.9	11.6	0.578
	Female	62.2	16.2		85.5	11.9	
**Qualification**	Diploma	61.8	13.9	0.970	85.5	11.2	0.001*
	Degree	61.7	19.4		80.1	12.3	
**Service year**	0–3	62.5	16.0	0.987	83.4	11.8	0.904
	4–7	57.8	15.6		85.4	11.3	
	8–11	63.9	10.0		86.5	11.2	
	2–15	59.5	16.3		88.0	10.4	
	> = 20	67.6	18.9		81.4	14.7	
**Previous training**	Yes	62.5	16.6	0.461	85.2	12.1	0.129
	No	61.1	14.4		83.3	11.4	
**Health institution**	Government	62.4	16.4	0.126	83.8	12.1	0.354
	Private	59.8	12.0		85.2	10.2	

SD = standard deviation, * = statistically significant.

**Table 4:** Pre and post training practical mean score on smear reading by different characteristics of trainees, Adama regional laboratory, 2009

**Trainees’ characteristics**		**Practical pre-training**			**Practical post- training**		
		**Mean score in %**	**SD**	**P-vale**	**Mean score in %**	**SD**	**P-value**
**Sex**	Male	75.2	13.9	0.386	89.1	10.8	0.986
	Female	78.3	15.6		89.9	11.3	
**Qualification**	Diploma	76.0	13.5	0.134	90.3	10.2	0.008*
	Degree	74.7	16.4		86.2	12.1	
**Service year**	0–3	75.8	15.0	0.396	88.7	11.3	0.737
	4–7	76.8	12.9		91.8	8.7	
	8–11	72.5	12.5		87.5	11.8	
	2–15	73.8	11.7		90.8	6.2	
	> = 20	83.2	11.7		93.8	8.5	
**Previous training**	Yes	84.7	10.0	<0.0001*	91.9	10.9	<0.0001*
	No	67.9	12.7		87.0	10.3	
**Health institution**	Government	75.9	15.3	0.660	88.5	11.3	0.011*
	Private	75.2	10.6		91.7	9.1	

SD = Standard deviation, * = statistically significant.

### Type of errors committed by trainees

Pre and post training score analysis on type and error rates has shown that majority of trainees committed quantification error but the percentage of trainees with this type of error substantially decrease after they were trained (p = 0.02). Before the training 12.4% of trainees were found to commit minor error of low false negative but after training, the percentage of trainees with this error were found to decrease (p < 0.0001) (Table 4). Similarly, the microscopic reading result of 5.5% of the trainees was found to be high false positive before trainings however after the training only 1% of participants were found to commit high false positive result (p = 0.031). Before and after the whole rounds of trainings, it was also observed that 9% and 35% of trainees respectively, have correctly read all slides (P < 0.0001) (Table 5).

**Table 5:** Distribution of trainees by microscopic smear reading error type before and after training, Adama regional laboratory, 2009

**Error type**	**Before training in %**	**After training in %**	**p-value**
**Quantification error**	66.2	57.0	0.020*
**Low false negative**	12.4	1.4	<0.0001*
**Low false positive**	3.4	3.3	0.549
**High false negative**	3.5	2.3	0.062
**High false positive**	5.5	1.0	0.031*
**No error**	9.0	35.0	<0.0001*

* = statistically significant.

### Error rates by trainee’s characteristics

Analysis of effect of trainees’ characteristics on error rates was carried out after categorizing all types of errors to one group (error), and correct reading in to no error. Accordingly, more males (95%) than females (92.1%) were found to commit at least one error in microscopic reading before training, but the difference was not statistically significant (p = 0.208). Except participation in previous training, other trainees’ characteristics were also not associated with smear reading errors. Trainees who have participated in previous training than those who have not, were found to read all slides without error (p < 0.0001) (Table 6).

**Table 6:** Pre and post training error by characteristics of trainees, Adama regional laboratory, 2009

**Trainees’ characteristics**		**Pre training error**			**Post training error**		
		**(n = 316)**			**(n = 316)**		
		**Yes n(%)**	**No n(%)**	**P-value**	**Yes n(%)**	**No n(%)**	**P-value**
		**N (%)**	**N (%)**		**N (%)**	**N (%)**	
**Sex**	Male	246(95)	13(5)	0.208	179(69.1)	80(30.9)	0.064
	Female	52(91.2)	5(8.8)		32(56.1)	25(43.9)	
**Qualification**	Diploma	227(95.4)	11(4.6)	0.125	152(69.9)	86(36.1)	0.071
	Degree	71(91.0)	7(9.0)		59(75.6)	19(24.4)	
**Service year**	0–3	196(94.7)	11(5.3)	0.492	143(69.1)	64(30.9)	0.280
	4–7	58(92.1)	5(7.9)		38(60.3)	25(37.7)	
	8–11	36(97.3)	1(2.7)		25(67.6)	12(32.4)	
	12–15	4(100)	0(0)		3(75.0)	1(25.0)	
	> = 20	4(80)	1(20)		2(40.0)	3(60.0)	
**Previous training**	Yes	130(88.4)	17(11.6)	<0.0001*	75(51.0)	72(49.0)	<0.0001*
	No	168(99.4)	1(0.6)		136(80.5)	33(19.5)	
**Health institution**	Government	219(92.8)	17(7.2)	0.051	164(77.7)	47(23.3)	0.099
	Private	79(98.8)	1(1.2)		72(68.6)	33(31.4)	

* = statistically significant.

## Discussion

In 2009, eight rounds of trainings were conducted for 316 laboratory professionals with objective to strengthen AFB microscopy service in Oromia region. Data on those trainings were retrospectively investigated to measure the effect of training on performance of trainees in the form of post test assessment. Most of the trainees were diploma graduates with maximum service year of 20 years. On the contrary, those participants with qualification of BSc degree have minimal service years and this may be due to the fact that the BSc degree program in Ethiopia has started recently. Nearly half of the trainees enrolled in the current investigation have also had similar training before.

Training along with other interventions is very important to strength AFB microscopy centers [13]. The purpose of training is to improve the performance of professionals and this requires analysis of training data as it is important for planning. Our investigation on training data has revealed that trainees have significantly improved their performance both in theoretical knowledge and practical skills. The average pre training proficiency of trainees on smear reading was found to be 75.6% but it was increased to 89% after the training. In routine panel testing, participants are expected to score a proficiency of 80% in smear reading [16]. Proficiency of the trainees was lower than the standard before they were trained but due to the effect of training they were able to attain the standard score. Studies in other countries have also reported similar findings [9,10,14]. Besides improving the proficiency, training can also motivate, update on new information and facilitate ways to share experience so that laboratory professionals can thrive for better services.

In routine AFB microscopy set ups, laboratory professionals have different back grounds in terms of qualification, training, experience and others. Therefore it is important to consider all these characteristics for possible interventions during training as well as analysis of training data. In the current investigation, post training theoretical performance of laboratory professionals with qualification of BSc degree was lower than the diploma graduates. In principle, the performance of BSc degree graduates is expected to be better than diploma graduates. The observed difference may be due to lack of attention resulting from over confidence by BSc degree graduates during trainings.

In pre and post practical assessment, trainees who have participated in previous similar training have shown better performance than those who have not been trained previously. This finding indicates that training and re-training is important to improve practical performance of laboratory professionals. Other studies have also reported the importance of on job training in improving performance of laboratory professionals involved in AFB microscopy [9,13,15]. In addition to training, it is also important to implement sustainable EQA program to have efficient AFB microscopy centers [13,17]. In the post training practical assessment, it was also observed that participants from private health facilities have performed better than those from government (p = 0.011). This could be due to the fact that trainees from private health facilities might have participated with more attention to achieve better to cope up with strict rules and regulations in private health facilities.

In routine AFB microscopy, both minor (QE, LFN, and LFP) and major (HFN, HFP) errors occur at a different rates as a result patient management as well as TB control program can be affected depending on the magnitude and type of error. Our investigation has identified higher rate of QE which is comparable with other study in Mexico [13] but study in India has reported higher rate of LFN than QE [15]. In our study, significant number of participants had previous similar training, but participants in the study of India were fresh graduates. This could be the possible reason for the observed variation.

Proportion of trainees who committed false positive (LFP and HFP) and negative (LFN and HFN) errors were significantly reduced after they were trained, but these errors are not totally avoided. Data from other studies [9,10,15] have also shown similar finding. This could be due to the inherent low sensitivity of Ziehl-Neelsen AFB microscopy, technical problems which can be tackled through continuous refresher training and other interventions. In routine EQA, any major error (HFP or HFN) or any HFP with more than three LFN is not acceptable performance, but lower rates of minor error can be acceptable if the numbers do not demonstrate trends. Unlike QE, false negative and false positive errors have significant impact on patient management as well as the TB control program [16]. Hence, improving the competency of professionals’ thorough refresher training, implementation of EQA and other interventions are critically important to reduce or avoid these types of errors.

In our investigation, it was found that participants who have participated in previous similar trainings were found to commit fewer errors than those who had no previous training. This finding may indicate the actual performance in facilities. Ideally, it is important to evaluate the impact of training on performance of microscopy centers. A post training evaluation on impact of AFB microscopy training in Ghana has reported better performance of AFB microscopy centers [9]. The successive improvement of case detection in Oromia region from previous 32% to current 39% (unpublished report) indicates the improved performance of AFB microscopy centers and this could be due continuous refresher trainings and other interventions.

Our study was not without limitations. The main pitfall of our study was the fact that it failed to conduct impact assessment at each health facility where the trainees were based after the training on case detection due to financial constraints.

## Conclusions

In conclusion, training has improved theoretical and practical performance of laboratory professionals. Training has reduced minor error (QE, LFN, and LFP) and major error (HFN and HFP) but it has not totally avoided these errors. Pre-placement and continuous training irrespective of laboratory professionals’ qualification and service year, and sustainable EQA are highly recommended to ensure quality AFB microscopy service.

## Competing interests

The authors declare that they have no competing interests.

## Authors’ contributions

PR was involved conception and design of the study, data analysis and write up; GA was involved in data collection and analysis; GAbebe was involved in data analysis and reviewed the paper critically. All the authors read the final paper and approved it.

## Pre-publication history

The pre-publication history for this paper can be accessed here:

http://www.biomedcentral.com/1472-6963/13/392/prepub
